# Parallel evolution of storage roots in morning glories (Convolvulaceae)

**DOI:** 10.1186/s12870-018-1307-4

**Published:** 2018-05-29

**Authors:** Lauren A. Eserman, Robert L. Jarret, James H. Leebens-Mack

**Affiliations:** 1 Plant Biology Department, University of Georgia, Athens, GA 30602 USA; 2 U.S. Department of Agriculture, Plant Genetic Resources Conservation Unit, Griffin, GA 30223 USA; 3 Present address: Conservation and Research Department, Atlanta Botanical Garden, Atlanta, GA 30309 USA

**Keywords:** Comparative transcriptomics, Gene expression, *Ipomoea*, *Ipomoea batatas* (sweetpotato), Parallel evolution, Root anatomy, Storage roots

## Abstract

**Background:**

Storage roots are an ecologically and agriculturally important plant trait that have evolved numerous times in angiosperms. Storage roots primarily function to store carbohydrates underground as reserves for perennial species. In morning glories, storage roots are well characterized in the crop species sweetpotato, where starch accumulates in storage roots. This starch-storage tissue proliferates, and roots thicken to accommodate the additional tissue. In morning glories, storage roots have evolved numerous times. The primary goal of this study is to understand whether this was through parallel evolution, where species use a common genetic mechanism to achieve storage root formation, or through convergent evolution, where storage roots in distantly related species are formed using a different set of genes. Pairs of species where one forms storage roots and the other does not were sampled from two tribes in the morning glory family, the Ipomoeeae and Merremieae. Root anatomy in storage roots and fine roots was examined. Furthermore, we sequenced total mRNA from storage roots and fine roots in these species and analyzed differential gene expression.

**Results:**

Anatomical results reveal that storage roots of species in the Ipomoeeae tribe, such as sweetpotato, accumulate starch similar to species in the Merremieae tribe but differ in vascular tissue organization. In both storage root forming species, more genes were found to be upregulated in storage roots compared to fine roots. Further, we find that fifty-seven orthologous genes were differentially expressed between storage roots and fine roots in both storage root forming species. These genes are primarily involved in starch biosynthesis, regulation of starch biosynthesis, and transcription factor activity.

**Conclusions:**

Taken together, these results demonstrate that storage roots of species from both morning glory tribes are anatomically different but utilize a common core set of genes in storage root formation. This is consistent with a pattern of parallel evolution, thus highlighting the importance of examining anatomy together with gene expression to understand the evolutionary origins of ecologically and economically important plant traits.

**Electronic supplementary material:**

The online version of this article (10.1186/s12870-018-1307-4) contains supplementary material, which is available to authorized users.

## Background

Parallel and convergent evolution of complex morphological traits has long been of interest to evolutionary biologists, who have noted that functionally and morphologically similar phenotypes have evolved independently in unrelated lineages. Studies characterizing the genetic basis of independent phenotypic evolution have concluded that many traits evolve convergently, appearing phenotypically and functionally similar but utilizing different genetic mechanisms e.g. [1–3]. Alternatively, traits evolving in parallel have the same genetic basis [4–6]. Often, differentiating between these alternative evolutionary scenarios is difficult. Studies comparing morphology, anatomy, gene expression and other aspects of a trait can provide insights into whether a trait evolved convergently or in parallel.

Morning glories offer an ideal system in which to address hypotheses regarding convergent versus parallel evolution. In morning glories, storage root formation has been either lost or gained at least ten times independently, and storage roots are found in many diverse morning glory lineages such as those containing *I. batatas*, *I. lindheimeri*, and *Distimake dissectus*; however, it is unclear whether the ancestor of all morning glories was able to form storage roots [7]. Studies characterizing storage root development in sweetpotato have demonstrated that a storage root is simply a modification of the taproot, an adventitious root, and/or one or more lateral roots such that the root cambium expands and the starch-storage tissue proliferates [8–12]. The proliferation of starch-storage tissue expands the root so that storage roots are much greater in diameter than roots which do not function in long-term starch storage. Studies analyzing gene expression differences between fine and storage roots in sweetpotato (*Ipomoea batatas*) have found that genes in the starch biosynthesis pathway are highly expressed and lignin biosynthesis genes have reduced expression in storage roots compared to fine roots [8]. Studies have also implicated three genes in the development of storage roots, two of which are MADS-box transcription factors [13, 14] and the other is an alpha-expansin gene [15]. However, these studies were strictly limited to sweetpotato. Comparative studies may reveal genes involved in storage root formation across distantly related species.

In addition to the evolutionary importance, storage roots have economic and ecological significance as well. Sweetpotato [*Ipomoea batatas* (L.) Lam.] ranks among the ten most important crop species for human nutrition. In 2014, over 100 million tonnes of sweetpotato were produced worldwide [16]. The large storage roots are an important source of carbohydrates and vitamin A in developing countries [17]. More generally, storage roots play a key role in the life history and ecological strategies of plants, as perennial species tend to mobilize starch to roots year-round and thus form storage roots but annual species cease starch mobilization after only a few months [18]. Additionally, root carbohydrate reserves are necessary for resprouting after cutting or large-scale events such as fire [19–21].

Given what is known about the developmental biology and anatomy of storage roots, lineages that form storage roots may represent instances of either convergent or parallel evolution. In this study, we aim to: 1) to understand the anatomy of storage roots in morning glories and 2) to characterize gene expression during an early stage of storage root formation. If we observe that storage roots from distantly related morning glory lineages are anatomically similar and share an overlapping set of differentially expressed orthologous genes, this would provide evidence supporting the hypothesis that storage roots evolved prior to the diversification of morning glories and were subsequently lost in lineages that do not form storage roots (parallel evolution). However, if we observe that storage roots are anatomically dissimilar and share few to no differentially expressed orthologous genes, this would support the hypothesis that storage roots evolved independently in storage root forming lineages (convergent evolution). Using this comparative approach, we can better understand the genetic mechanisms and evolutionary origins of storage root formation. Through this work we ultimately seek to understand the genetic basis of storage root formation and whether independent lineages utilize the same or different genetic mechanisms during storage root development across the morning glory phylogeny.

## Methods

### Plant material

Three pairs of closely-related species were selected from across the morning glory phylogeny, where one member of the species pair produces storage roots and fine roots and the other produces only fine roots. The three storage root forming species are *Ipomoea batatas* (L.) Lam. (sweetpotato), *I. lindheimeri* A. Gray and *Distimake dissectus* (Jacq.) Simoes & Staples (formerly *Merremia dissecta*), and the species that produce only fine roots are *I. trifida* G. Don, *I. nil* (L.) Roth, and *D. quinquefolius* (L.) Simoes & Staples (formerly *Merremia quinquefolia*). All three pairs of species were utilized for anatomical observations. Four species, *I. batatas*, *I. trifida*, *D. dissectus* and *D. quinquefolius*, were used for transcriptome sequencing so that we could directly contrast gene expression of different observed root architectures. Plant material was obtained from outside sources, including USDA GRIN, seed companies, and the seed collections of R. Miller and J. Ekrut (Additional file 1: Table S1). Three biological replicates were chosen for each species except *I. trifida*, where RNA-seq libraries for one sample consistently failed. Sweetpotato (*I. batatas*) is hexaploid, and *I. trifida* is diploid [22]. Ploidy of *D. dissectus* and *D. quinquefolius* have not been previously determined; therefore, we attempted root tip squashes but were not able to get separation among chromosomes for counting. Genome size estimates are often used to infer ploidy in some species; however, because chromosome number has never been determined in these species and genome size varies widely among morning glories of the same ploidal level [22–24], we are not able to use this method to determine ploidy.

Sweetpotato is vegetatively propagated, so cuttings were planted of the three cultivars (Beauregard, Jewel, and Tinian) with three true leaves. Seeds of the other five species were scarified before planting. Seeds and cuttings were planted in Fafard 3B mix in 4″ square pots. Seeds were allowed to germinate for 1 week in the UGA Greenhouses. Plants were then moved to a growth chamber under an 8 h photoperiod and 30°/25 °C day/ night temperatures [14]. Previous studies have found that storage root formation occurs within four to six weeks after planting in sweetpotato [8, 25]; therefore, plants in this study were grown for six weeks prior to sampling. Roots were sampled using the following procedure: roots were removed from medium, washed in tap water, and rinsed a final time in nuclease-free molecular biology grade water. The primary root was dissected from the whole plant, and fine lateral roots were then dissected from the primary root. Fresh root tissue was flash frozen in liquid nitrogen and was subsequently stored at − 80 °C until RNA isolation. Alternatively, fresh root tissue was used immediately for anatomical observations.

### Anatomical observations

Fresh root tissue was sectioned by hand with a razor blade. A main goal of this was to observe the spatial deposition of starch in cross sections of the root; therefore, fresh sections were necessary because starch is removed during standard tissue clearing [26]. Serial sections were taken from fine roots and from two places on the taproot or storage root: 1) after the 4th lateral root, and 2) after the 10th lateral root. Sections were stained with Lugol’s iodine, a solution of iodine and potassium iodide which indicates the presence of starch, or phloroglucinol-HCl, which stains lignin [27], immediately following sectioning. Stained sections were mounted in a filtered 20% CaCl_2_ solution [28]. Mounted sections were viewed with a Zeiss Axio microscope with attached camera under either a 2.5× or 10× objective lens. Sections too large to be viewed in a single field of vision using the 2.5× objective lens were captured in multiple images which were then stitched together using the image stitching plugin for the Fiji distribution of ImageJ [29–31]. Field of vision length was determined using a standard microscope scale, and scale bars were added to images in ImageJ.

### RNA isolations and library construction

Total RNA was isolated from frozen root tissue using the standard Trizol protocol (Life Technologies). RNA was eluted in molecular biology grade H_2_0 following isolation. DNA was removed using the TURBO DNA-free kit (Thermo Fisher Scientific). Prior to library construction, RNA quality was assessed with the Agilent Bioanalyzer 2100 using the RNA 6000 Nano kit (Agilent Technologies, Santa Clara, CA). mRNA was isolated from total RNA using the NEBNext Poly(A) mRNA Magnetic Isolation Module (New England Biolabs, Inc.). The first mRNA isolations performed using the recommended total RNA input yielded low mRNA concentrations. Therefore, the amount of total RNA added to the mRNA isolation protocol was increased to 5 μg, the maximum recommended RNA input. Libraries were constructed with the NEBNext Ultra Directional RNA Library Prep Kit for Illumina (New England Biolabs, Inc.) using the standard protocol with slight modifications. Libraries were amplified with 15 PCR cycles. An initial test set of libraries showed adapter dimer peaks; therefore, the adapter was diluted 1.25 μM rather than the standard 1.5 μM, which eliminated adapter dimer peaks in future libraries. The library preparation protocol used in this experiment implements the dUTP method [32] to generate stranded libraries.

Libraries were quantified using quantitative real-time PCR prior to sequencing. Libraries were diluted to 10 nM for sequencing. Barcoded and diluted libraries were pooled before sequencing. All libraries were sequenced at the Georgia Genomics Facility on the Illumina NextSeq platform with paired-end 150 bp reads. Illumina sequence data used to assemble transcriptomes has been deposited to the GenBank Sequence Read Archive database under Bioproject PRJNA448837.

### Transcriptome analysis

Reads for each species were assembled separately into transcripts with the Trinity software suite version r20140717 [33]. Within-species transcriptome assembly and analysis followed the developed Trinity pipeline [33, 34]. Read quality was assessed with FastQC. Prior to assembly, reads were quality trimmed with Trimmomatic as implemented in the Trinity package. Bases at the beginning and end of a read with a phred score less than 5 were removed. In addition, reads less than 50 bp long were removed. Reads for each library were digitally normalized to a maximum of 50× coverage within Trinity (−-normalize_reads) to accelerate the assembly process. Reads were considered paired-end in the assembly, where the first read of the pair was considered the reverse read and the second was the forward read (−-SS_lib_type RF).

We then filtered assemblies to remove poorly supported isoforms and contaminants. We used RSEM version 1.2.20 [35] to estimate gene and transcript abundances as implemented in the Trinity package (align_and_estimate_abundance.pl script). Non-normalized reads were mapped to each transcriptome assembly with Bowtie 2 [36]. Isoforms which were supported by less than 30% of the total reads for a gene from two or more biological replicates or had an FPKM less than 2 were removed, as these represent possible assembly artifacts. Filtering was performed using the perl script filter_fasta_by_rsem_values.pl in the Trinity software package [34]. To remove contaminants, we annotated the assembled transcriptomes in Trinotate [34] using a blastx of the filtered assembly against the Uniprot database. Transcripts with annotations from any taxon other than Viridiplantae with an e-value greater than 1e-5 and 40% identity were removed as potential contaminants. Finally, the program DeconSeq version 0.4.2 [37] was used to further filter any remaining bacterial, viral, and human contaminant sequences.

RSEM [35] and Bowtie 2 [36] were again used to map reads from individual libraries back to the filtered transcriptome assemblies and calculate transcript abundances. EdgeR [38] was then used to assess differentially expressed genes between storage roots and fine roots of sweetpotato and *Distimake dissectus* using perl scripts from the Trinity analysis pipeline [34]. EdgeR was run separately for each species and incorporated biological replicates for each tissue type. FPKM values for each library were normalized by library size. This normalization process is referred to as “Trimmed Mean of M-values”, or TMM, normalization [39]. Only TMM-normalized FPKM values were used for differential expression analysis (Additional file 2: Table S2, Additional file 3: Table S3, Additional file 4: Table S4, and Additional file 5: Table S5). Transcripts were considered significantly differentially expressed at a false discovery rate (FDR) less than 0.05 and a log fold change of 2 (Additional file 6: Table S6 and Additional file 7: Table S7). We then generated Euclidean distances among transcripts and libraries and used a complete linkage clustering approach on the Euclidean distance matrices to cluster transcripts and libraries in edgeR.

Protein coding regions were identified from the final filtered assemblies using the program Transdecoder [34]. Protein sequences shorter than 50 amino acid residues long were not kept in the final set of peptide sequences. Functional annotation utilized the standard Trinotate pipeline [34], which incorporated a blastx search of the assembled transcripts against the Uniprot database and a blastp search of the peptide sequences inferred from Transdecoder against the Uniprot database. These results as well as gene ontology (GO) term annotations of the best gene match in Uniprot were incorporated into a SQLite database using Trinotate [34] (Additional file 8: Table S8, Additional file 9: Table S9, Additional file 10: Table S10, and Additional file 11: Table S11).

Peptide sequences from the final filtered assemblies from all four species were sorted into gene families with OrthoFinder [40] to determine orthology among transcripts from the four species. Coding sequences of the gene families estimated from OrthoFinder were aligned in SATé-II [41]. Gene trees were estimated in RAxML [42], and node support was determined using 500 bootstrap replicates.

## Results

### Root anatomy

**Fig. 1:**
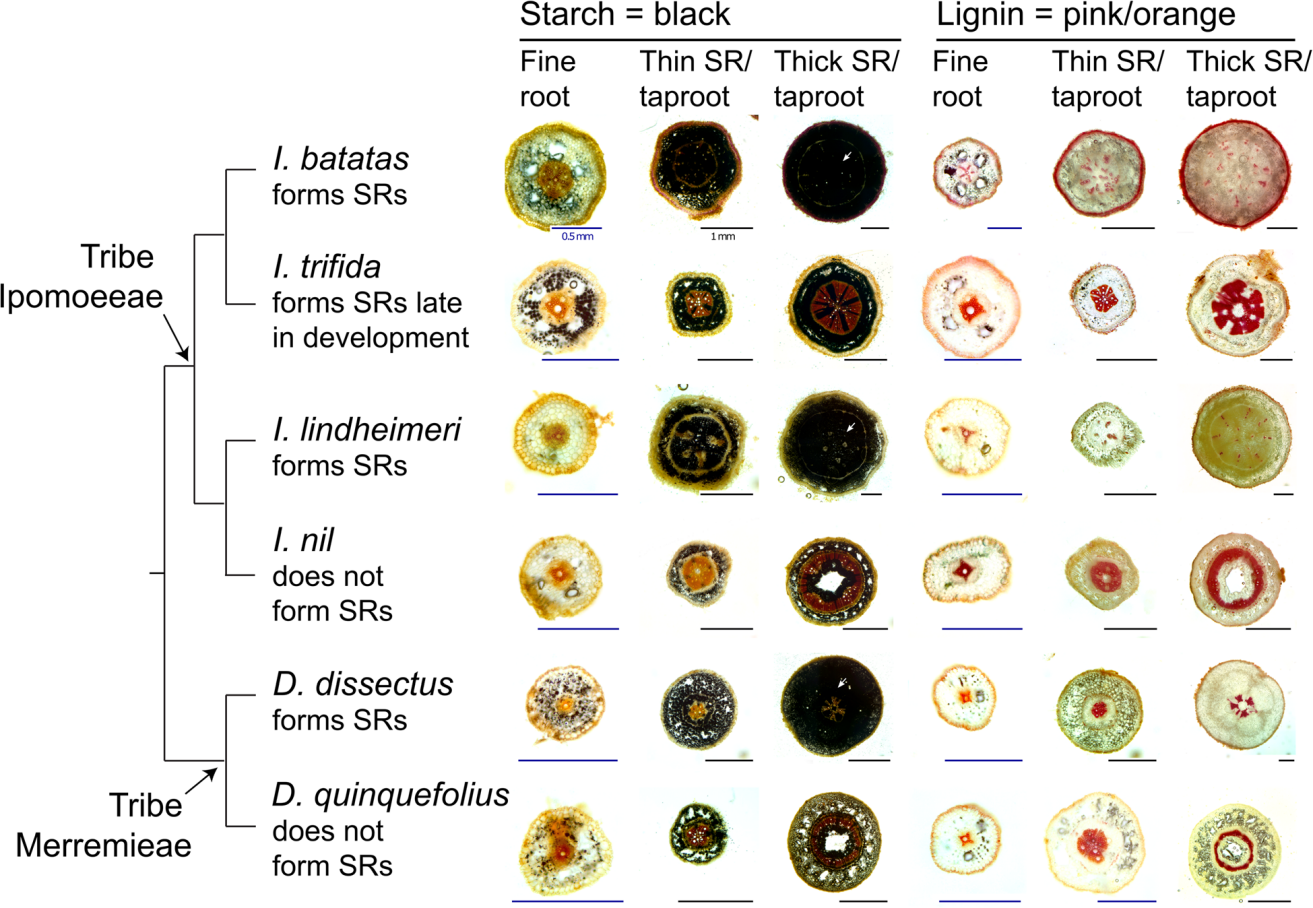
Root cross sections from three pairs of species, where one member of the species pair forms storage roots and the other does not. To the left is a phylogeny depicting the evolutionary relationships among the six species with arrows denoting the two tribes, Ipomoeeae and Merremieae. The left-most three columns are root sections stained with Lugol’s iodine, which indicates starch a dark blue to black color. The right-most three columns are root sections stained with phloroglucinol-HCl, which stains lignin orange to pink. Scale bars are included with each section. Black bars are 1 mm, and blue bars are 0.5 mm in length. White arrows indicate starch-storage tissue

Results of the root anatomical observations are shown in Fig. 1. There were three main results from this. First, fine roots of all six species are anatomically similar and exhibit the typical eudicot root anatomy with highly organized vascular tissue in the center and a larger cortex. Second, we found that the taproot of the species that do not form storage roots appear similar, accumulate very little starch, and do not show evidence of proliferation of starch-accumulating cells. Third, storage roots of the three storage root forming species showed similar starch accumulation, specifically, proliferation of the starch-accumulating cells that occurred within the bounds of the endodermis. Finally, the vascular tissue in storage roots of sweetpotato and *I. lindheimeri* appeared visually similar, where the starch-accumulating cells disrupted the organization of the vascular tissue. In contrast, the vascular tissue of storage roots of *Distimake dissectus* appeared markedly different such that vascular tissue was tightly organized in the center of the cross section.

### Transcriptome assembly statistics

The final dataset included seventeen RNA-seq libraries from two pairs of morning glory species. Transcriptome assembly statistics are shown in Table 1. Before filtering, the *Distimake quinquefolius* transcriptome had the largest number of transcripts, and the *I. trifida* transcriptome had the fewest assembled transcripts. Transcript N50 ranged from 952 to 1277 nt. We then filtered the raw assemblies by isoform percentage and FPKM, which resulted in a 42–70% reduction in the number of transcripts in the assembly (Table 2). This step removed potentially erroneous transcripts that were not supported by re-mapped reads. Further filtering of bacterial, fungal, algal, and viral transcripts using Swiss-prot annotations and DeconSeq resulted in an additional ca. 3900–5700 transcripts removed from each assembly. Only the transcriptomes filtered by isoform percentage and FPKM and which had contaminants removed were used for downstream analyses.

**Table 1:** Transcriptome assembly statistics

	*I. batatas*	*I. trifida*	*D. dissectus*	*D. quinquefolius*
Total reads (PE 150 bp)	39,632,572	15,657,942	44,877,650	64,267,290
No. of transcripts	245,140	119,153	254,174	363,820
%GC	40.97	42.1	39.67	39.37
Transcript N50	952	1125	1277	952
Median transcript length	416	455	446	417
Mean transcript length	663.57	732.7	777.18	663.29

**Table 2:** Assembly statistics after successive filtering by IsoPct and FPKM, Swiss-prot annotations, and Decon-Seq

	*I. batatas*	*I. trifida*	*D. dissectus*	*D. quinquefolius*
Transcripts in original assembly	245,140	119,153	254,174	363,820
Transcripts filtered by IsoPct, FPKM	158,267 (64.6%)	51,181 (43.0%)	176,584 (69.5%)	209,593 (57.6%)
Transcripts filtered by Swiss-prot annotations	5097 (2.1%)	5262 (4.4%)	3529 (1.4%)	3665 (1.0%)
Transcripts filtered by Decon-Seq	619 (0.3%)	491 (0.4%)	441 (0.2%)	595 (0.2%)
Total removed by filtering	163,983 (66.9%)	56,934 (47.8%)	180,554 (71.0%)	213,853 (58.8%)

### Within species differential gene expression

**Fig. 2:**
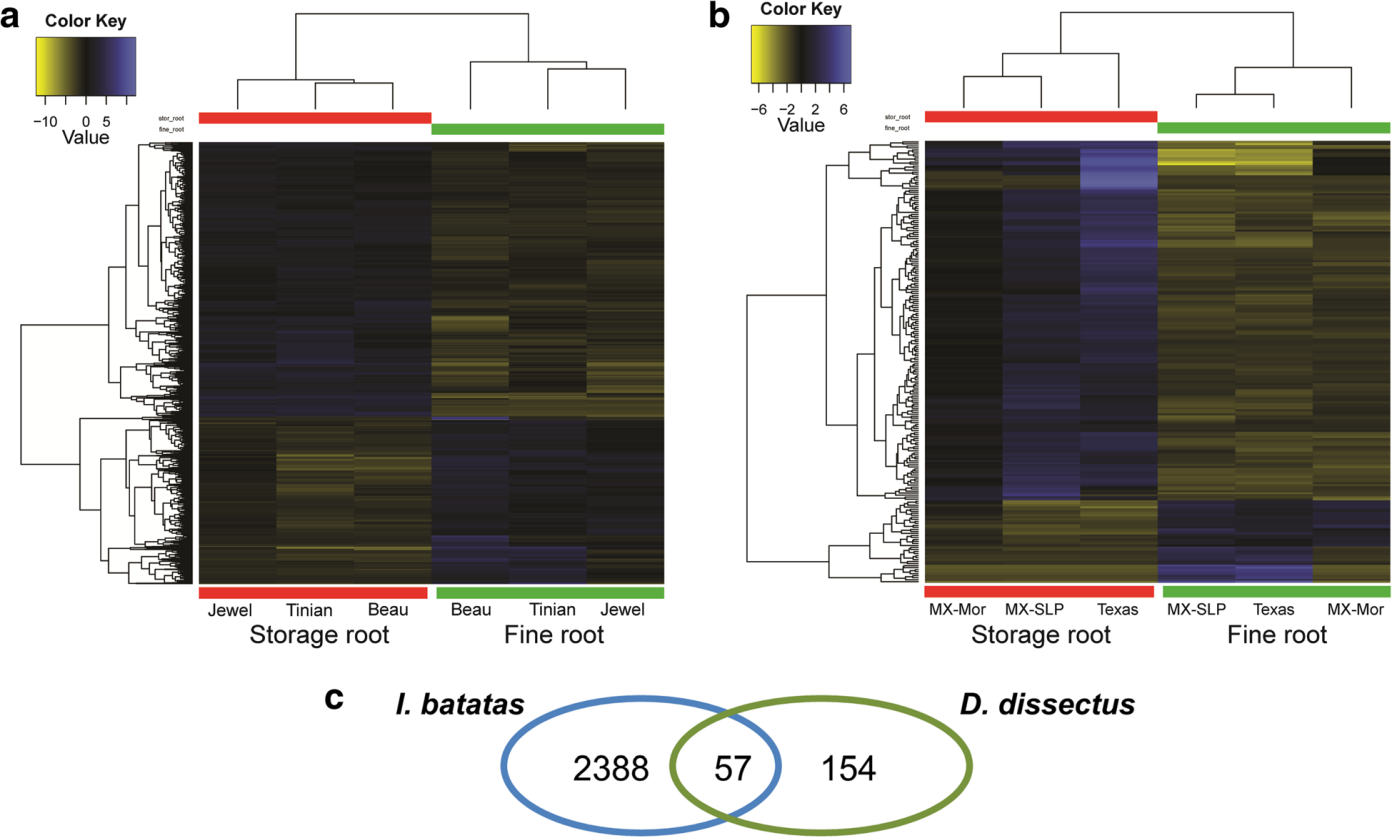
Heat map of genes differentially expressed between storage roots and fine roots of sweet potato, *Ipomoea batatas* (**a**) and *Distimake dissectus* (**b**). Each row in the heatmap is depicting the expression patterns of each transcript, and each column represents each library. A dendrogram illustrating clustering of libraries is shown above each heatmap, and a dendrogram showing clustering of transcript expression patterns is to the left of each heatmap. **c** Number of transcripts differentially expressed between storage and fine roots and the number that were orthologous between *I. batatas* and *D. dissectus*

We assessed differential gene expression between storage roots and fine roots in sweetpotato and *Distimake dissectus* separately. After accounting for multiple comparisons, there were 2643 genes differentially expressed (DE) between storage roots and fine roots in sweetpotato and 219 DE genes in *D. dissectus* at a FDR < 0.05 (Fig. 2a, b). In both species, there were more transcripts highly expressed in storage roots than in fine roots. As a convention, upregulated transcripts refers to those more highly expressed in storage roots vs. fine roots and downregulated refers to transcripts with reduced expression in storage roots compared to fine roots. In sweetpotato, 1642 transcripts were upregulated and 1001 transcripts were downregulated. In *Distimake dissectus*, there were 178 upregulated transcripts and 41 downregulated transcripts.

The top ten most abundant gene ontology annotations for the differentially expressed genes in sweetpotato and *D. dissectus* are found in Table 3. When we compare the ten most abundant GO annotations from genes DE in sweetpotato and *D. dissectus*, we find that eight of these GO terms overlap. Additionally, many of the most enriched GO terms were involved in transcription or are annotated as having transcription factor activity (Table 3).

**Table 3:** Top ten most abundant gene ontology (GO) categories represented in genes differentially expressed between storage roots and fine roots of *Ipomoea batatas* and *Distimake dissectus* considering each species separately

Species	% of Total	GO annotation	Type	Specific
*I. batatas*	4.50	GO:0016021	cellular_component	integral component of membrane
*I. batatas*	3.12	GO:0005634	cellular_component	nucleus
*I. batatas*	2.96	GO:0005886	cellular_component	plasma membrane
*I. batatas*	2.45	GO:0005524	molecular_function	ATP binding
*I. batatas*	1.98	GO:0046872	molecular_function	metal ion binding
*I. batatas*	1.89	GO:0006351	biological_process	transcription, DNA-templated
*I. batatas*	1.69	GO:0005576	cellular_component	extracellular region
*I. batatas*	1.68	GO:0009507	cellular_component	chloroplast
*I. batatas*	1.63	GO:0003700	molecular_function	sequence-specific DNA binding transcription factor activity
*I. batatas*	1.57	GO:0003677	molecular_function	DNA binding
*D. dissectus*	4.37	GO:0016021	cellular_component	integral component of membrane
*D. dissectus*	3.06	GO:0005634	cellular_component	nucleus
*D. dissectus*	2.51	GO:0005886	cellular_component	plasma membrane
*D. dissectus*	2.51	GO:0003700	molecular_function	sequence-specific DNA binding transcription factor activity
*D. dissectus*	2.18	GO:0006351	biological_process	transcription, DNA-templated
*D. dissectus*	2.07	GO:0005524	molecular_function	ATP binding
*D. dissectus*	1.86	GO:0009507	cellular_component	chloroplast
*D. dissectus*	1.53	GO:0006355	biological_process	regulation of transcription, DNA-templated
*D. dissectus*	1.53	GO:0003677	molecular_function	DNA binding
*D. dissectus*	1.42	GO:0009501	cellular_component	amyloplast

### Between species differential gene expression

To compare gene expression between orthologs of different species, we sorted transcripts into orthologous groups with OrthoFinder [40]. We then queried the orthologous groups for known sets of transcripts differentially expressed (DE) between storage and fine roots in sweetpotato and *Distimake dissectus*. We found there were 57 orthologous genes DE between storage roots and fine roots of both species (Fig. 2c). We then examined GO term annotations for the set of orthologous DE transcripts (Table 4). Transcripts annotated with amyloplast or starch biosynthetic activity were found to represent a larger percent of the total GO annotations in the set of shared DE transcripts than in the DE transcripts from sweetpotato and *D. dissectus* analyzed separately (Tables 3, 4). Similarly, we examined the functional annotation of these transcripts and found that some of these DE genes share close homology with transcription factors, alpha-expansin genes, genes that function in the starch biosynthetic pathway, and one that functions in the starch degradation pathway.

**Table 4:** Top ten most abundant gene ontology (GO) categories represented in the set of orthologous genes differentially expressed between storage roots and fine roots in both *Ipomoea batatas* and *Distimake dissectus*

Species	% of Total	GO annotation	Type	Specific
*I. batatas*	3.89	GO:0009507	cellular_component	chloroplast
*I. batatas*	3.53	GO:0016021	cellular_component	integral component of membrane
*I. batatas*	3.18	GO:0009501	cellular_component	amyloplast
*I. batatas*	3.18	GO:0005634	cellular_component	Nucleus
*I. batatas*	2.83	GO:0005524	molecular_function	ATP binding
*I. batatas*	2.12	GO:0019252	biological_process	starch biosynthetic process
*I. batatas*	2.12	GO:0003700	molecular_function	sequence-specific DNA binding transcription factor activity
*I. batatas*	1.77	GO:0006351	biological_process	transcription, DNA-templated
*I. batatas*	1.77	GO:0005886	cellular_component	plasma membrane
*I. batatas*	1.77	GO:0003677	molecular_function	DNA binding
*D. dissectus*	4.00	GO:0009507	cellular_component	chloroplast
*D. dissectus*	4.00	GO:0016021	cellular_component	integral component of membrane
*D. dissectus*	3.20	GO:0009501	cellular_component	amyloplast
*D. dissectus*	3.20	GO:0005634	cellular_component	Nucleus
*D. dissectus*	2.40	GO:0005576	cellular_component	extracellular region
*D. dissectus*	2.40	GO:0005524	molecular_function	ATP binding
*D. dissectus*	2.40	GO:0003677	molecular_function	DNA binding
*D. dissectus*	2.00	GO:0019252	biological_process	starch biosynthetic process
*D. dissectus*	2.00	GO:0006351	biological_process	transcription, DNA-templated
*D. dissectus*	2.00	GO:0003700	molecular_function	sequence-specific DNA binding transcription factor activity

### Among species differential gene expression

**Fig. 3:**
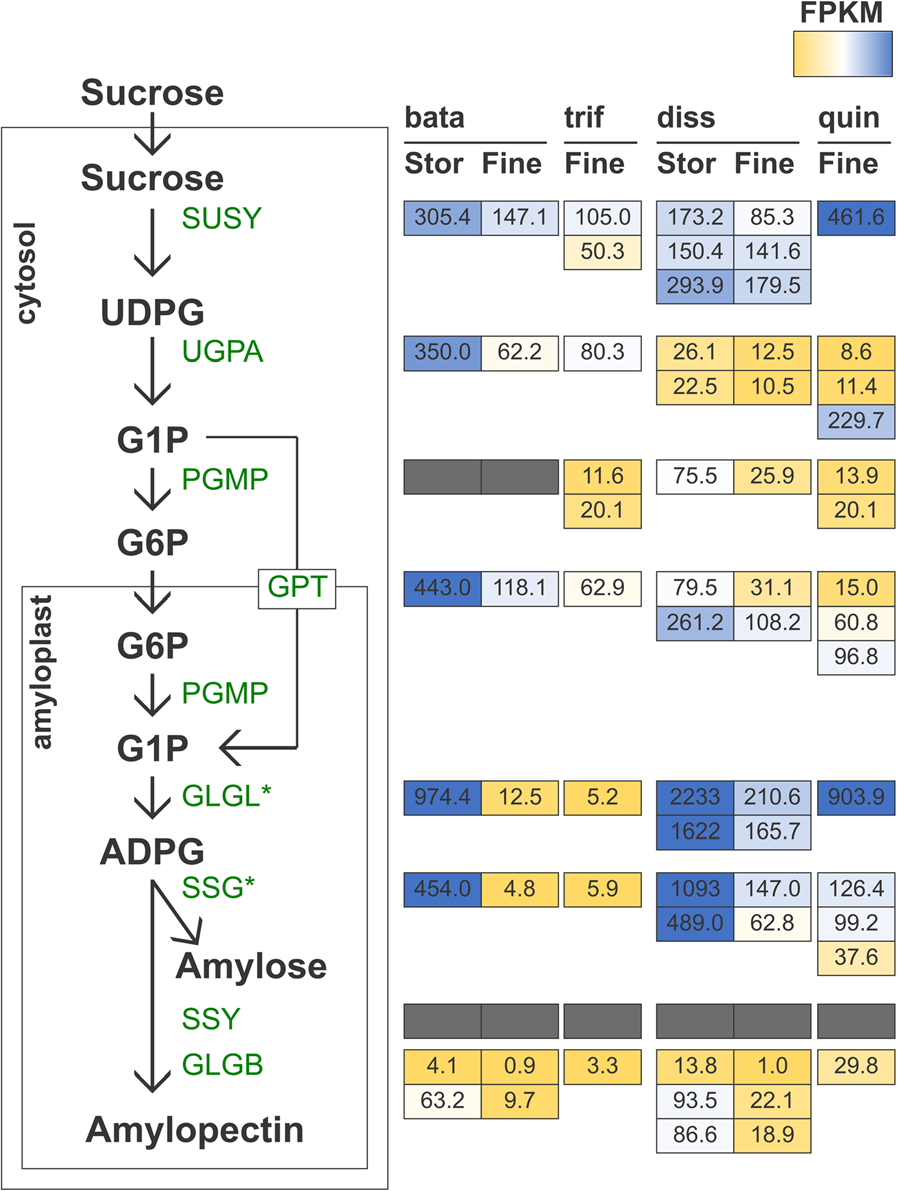
Starch biosynthetic pathway adapted from Bahaji et al. 2014. Metabolites are shown in black, and enzymes are shown in green. Shown are TMM-normalized FPKM values for homologs in all four species (bata = *Ipomoea batatas*, trif = *I. trifida*, diss = *Distimake dissectus*, and quin = *D. quinquefolius*). Grey boxes indicate genes where orthology could not be determined. Stacked boxes indicate homologs of a particular gene. Gene names with an asterisk were found to be significantly differentially expressed at a FDR < 0.05 in both *I. batatas* and *D. dissectus*. The heatmap is colored by percentile, where genes in the 10th percentile were colored yellow and those in the 90th percentile were colored dark blue

**Fig. 4:**
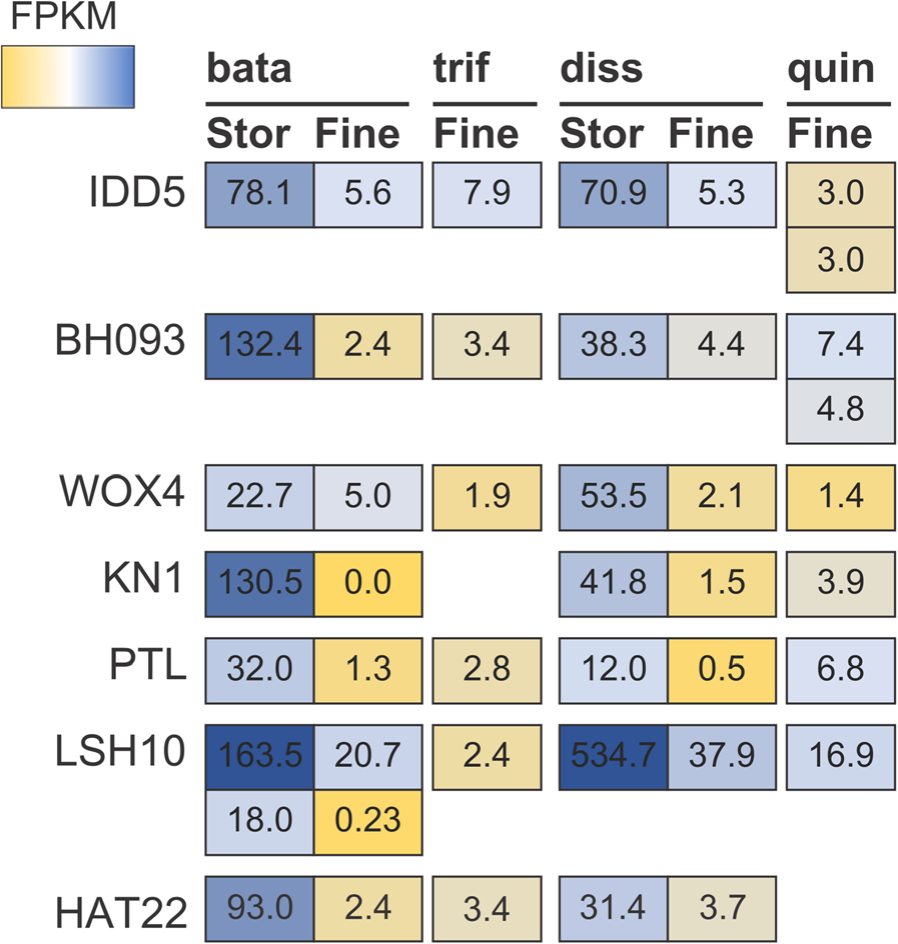
Mean TMM-normalized FPKM values for the seven transcription factors found to be significantly differentially expressed between storage and fine roots in both *Ipomoea batatas* and *Distimake dissectus* at a FDR < 0.05. The heatmap depicts mean TMM-normalized FPKM values for orthologs of the transcription factors in each tissue type for all four species (bata = *Ipomoea batatas*, trif = *I. trifida*, diss = *Distimake dissectus*, and quin = *D. quinquefolius*). The heatmap was colored by percentile, where genes in the 10th percentile were colored yellow and those in the 90th percentile were colored dark blue. No ortholog of KN1 could be identified in the transcriptome assembly of *I. trifida*, and no ortholog of HAT22 could be identified in *D. quinquefolius*

We then wanted to examine the expression of genes in the starch biosynthetic pathway (Fig. 3). Most genes in the starch biosynthesis pathway were found to have reduced expression. However, orthologs of GLGL1 and SSG1 were significantly differentially expressed in sweetpotato and *Distimake dissectus* (Fig. 3). These genes were highly expressed in storage roots and had reduced expression in fine roots, except for GLGL1 in *D. quinquefolius* (Fig. 3). Furthermore, we examined expression of transcripts annotated as having transcription factor activity, where orthologs were differentially expressed in both sweetpotato and *D. dissectus* (Fig. 4). In all cases, orthologs of the shared differentially expressed transcription factors were more highly expressed in storage roots than fine roots (Fig. 4).

## Discussion

### Root anatomy

Results of the root anatomical work clearly show that the storage roots of species in the tribe Ipomoeeae (sweetpotato and *I. lindheimeri*) are anatomically quite different from storage roots of *Distimake dissectus*, a member of the sister tribe Merremieae. Starch-accumulating cells proliferated in all three storage root forming species; however, xylem organization differed greatly in *D. dissectus* compared to storage roots of the other two species (Fig. 1). Our findings are consistent with other studies examining root anatomical structure of sweetpotato [8–12]. However, we had no a priori expectations with regard to root anatomy of all other species included in this study, as this is the first to document root anatomy of *I. lindheimeri*, *I. nil*, *I. trifida*, *D. dissectus*, and *D. quinquefolius*.

### Comparison of gene expression in all species

Based on the anatomical results, we can generate expectations with respect to the transcriptome experiment. Starch accumulation occurred similarly in storage roots of all three species; however, xylem organization was quite different in storage roots of *D. dissectus*. Therefore, it is likely that genes involved in starch biosynthesis and cell proliferation will be differentially expressed between storage and fine roots in both species, but genes involved in xylem organization may not show the same gene expression patterns between species.

At a broad level, more genes were found to be upregulated in storage roots compared to fine roots in both sweetpotato and *D. dissectus*. Interestingly, this result is in contrast to a previous RNA-seq study in sweetpotato which found an approximately equal number of genes up- and downregulated in storage roots compared to fine roots [8]. We sampled roots six weeks after planting in contrast to Firon et al. (2013), which sampled roots at four weeks. Given that we are sampling at a slightly later growth stage, perhaps we are capturing a more active stage of storage root bulking in this study. In the future, closer examination of the anatomical and gene expression changes during the very early stages of storage root formation would provide further insights into the development of this trait.

### Starch biosynthetic pathway

Starch biosynthesis occurs as part of a complex and dynamic pathway and the enzymes and transport proteins involved depend heavily upon the tissue in which starch is being synthesized. The process differs in photosynthetic and heterotrophic tissues [43]. Therefore, we focused on the starch pathway that has been characterized in potato tubers from Bahaji et al. [43] because it is the most well-characterized starch biosynthetic pathway in heterotrophic tissue in a species closely related to sweetpotato.

Whereas in photosynthetic tissue sucrose is broken into fructose and glucose prior to starch synthesis, in heterotrophic tissues, sucrose is directly converted to UDP-glucose before starch biosynthesis [43]. In addition, the downstream conversion of UDP-glucose to starch intermediates differs between eudicot and monocot heterotrophic tissues. UDP-glucose is converted to glucose-1-phosphate by the enzyme UDP-glucose pyrophosphorylase (UGPA). Glucose-1-phosphate is then either transported from the cytosol into the amyloplast or converted in the cytosol to glucose-6-phosphate by the enzyme phosphoglucomutase (PGMP). Glucose-6-phosphate is then transported into the amyloplast by the transport protein glucose-6-phosphate translocator (GPT) where it is converted back to glucose-1-phosphate by PGMP. Glucose-1-phosphate is converted to ADP-glucose by the action of ADP-glucose pyrophosphorylase (GLGL), which requires an input of ATP. ADP-glucose is then converted to the main components of starch by granule-bound starch synthase (SSG) to generate amylose or starch synthase (SSY) and starch branching enzymes (GLGB) to generate amylopectin.

In the context of this study, we found that orthologs of two genes involved in starch biosynthesis had significantly higher expression in storage roots compared to fine roots in both sweetpotato and *Distimake dissectus* (Fig. 3). In this study, GLGL1 and SSG1 were significantly differentially expressed between storage roots and fine roots of sweetpotato and *D. dissectus* (Fig. 3). GLGL acts downstream in the pathway, directly upstream of SSG, which is involved in the synthesis of amylose [43]. Generally, amylose content in sweetpotato cultivars is high, ranging from 20 to 33% of total starch content [44, 45], much higher than in other starch-rich root and tuber crops such as cassava [46].

This examination must be taken with the caveat that starch accumulation and bulking may occur through different mechanisms in sweetpotato and potato. First, sweetpotato storage roots and potato tubers arise from different tissue types; storage roots from root tissue and tubers from stem tissue [47]. Furthermore, tuber formation in potato is controlled by a homologue of flowering locus T (SP6A), and the process of tuber initiation is dependent on photoperiod [47]. However, experimental evidence has demonstrated that sweetpotato storage root initiation occurs under both long and short day regimes [48]. Future functional genomic research involving sweetpotato and its close relatives is necessary to elucidate the exact mechanisms of starch biosynthesis and storage.

### Transcription factors

Of the fifty-seven orthologous genes differentially expressed between storage roots and fine roots, seven were annotated as having transcription factor activity (Fig. 4). When we further examine the annotated functions of these genes, two stand out as potential candidate regulators of storage root formation.

IDD5, also called RAVEN, has been shown to positively regulate starch synthase in *Arabidopsis thaliana* [49]. Additionally, IDD5 is part of a larger regulatory network that, among other functions, regulates spatial patterning of root tissue through asymmetric cell division [49–51]. Many members of the larger regulatory network to which IDD5 belongs were found to be differentially expressed between SRs and FRs in sweetpotato cv. Georgia Jet and Xushu [8, 52], suggesting a possible role of IDD5 and members of this regulatory network in storage root formation.

Similarly, WOX4 orthologs were DE between storage roots and fine roots of both sweetpotato and *D. dissectus*. This gene has been shown to play a critical role in vasculature proliferation and secondary growth in *Arabidopsis thaliana*, and functions specifically within the cambium of stems and roots [53, 54]. Perhaps this gene plays a role in the proliferation of starch-storage tissue that we observe in storage roots of sweetpotato and *D. dissectus.*

## Conclusions

The anatomical results suggested that storage roots differ from fine roots in starch content, deposition and vasculature patterning. As expected, we found significantly higher expression of genes involved directly in starch biosynthesis in both storage root forming species and increased expression of IDD5, a transcription factor known to regulate starch biosynthesis in *Arabidopsis* [49]. Similarly, we found significant upregulation of WOX4, a gene known to be involved in vasculature proliferation in *Arabidopsis* [53, 54]. Given the large number of orthologous genes DE between storage roots and fine roots, we hypothesize that there was a single origin of storage roots before the divergence of the morning glory tribes Ipomoeeae and Merremieae given that storage roots in the species examined are superficially anatomically different but store starch in a similar manner. To further support this hypothesis, we find that many of the same genes were differentially expressed between storage roots and fine roots in sweetpotato and *Distimake dissectus*. However, an alternative hypothesis, that storage roots evolved multiple times independently using the same genetic mechanisms, cannot be directly rejected by these results. Therefore, much more work must be done to test these hypotheses in a rigorous framework. The findings presented here present a first step in understanding the evolution and development of a plant trait that has received little attention to date but is economically and ecologically important. These results further demonstrate the power of comparative studies to understand the development of a trait and its evolution in a deeper way than to examine a single species.

## Additional files

Additional file 1: **Table S1.** Accession information for the plant material used in this experiment. (XLSX 10 kb)

Additional file 2: **Table S2.** TMM-normalized FPKM values for fine root samples in *Ipomoea batatas*. Statistics were output from edgeR analysis. (XLSX 4456 kb)

Additional file 3: **Table S3.** TMM-normalized FPKM values for fine root samples in *Ipomoea trifida*. Statistics were output from edgeR analysis. (XLSX 1833 kb)

Additional file 4: **Table S4.** TMM-normalized FPKM values for fine root samples in *Distimake dissectus*. Statistics were output from edgeR analysis. (XLSX 4129 kb)

Additional file 5: **Table S5.** TMM-normalized FPKM values for fine root samples in *Distimake quinquefolius*. Statistics were output from edgeR analysis. (XLSX 5130 kb)

Additional file 6: **Table S6.** Genes differentially expressed between storage roots and fine roots in *Ipomoea batatas*. Statistics from edgeR on transcripts found to be significantly differentially expressed between storage roots and fine roots in *Ipomoea batatas*. (XLSX 142 kb)

Additional file 7: **Table S7.** Genes differentially expressed between storage roots and fine roots of *Distimake dissectus*. Statistics from edgeR on transcripts found to be significantly differentially expressed between storage roots and fine roots in *Distimake dissectus*. (XLSX 23 kb)

Additional file 8: **Table S8.** Functional annotation of genes and transcripts in *Ipomoea batatas* from Trinotate. (XLSX 12149 kb)

Additional file 9: **Table S9.** Functional annotation of genes and transcripts in *Ipomoea trifida* from Trinotate. (XLSX 10975 kb)

Additional file 10: **Table S10.** Functional annotation of genes and transcripts in *Distimake dissectus* from Trinotate. (XLSX 13051 kb)

Additional file 11: **Table S11.** Functional annotation of genes and transcripts in *Distimake quinquefolius* from Trinotate. (XLSX 23712 kb)

## Abbreviations

DE: Differentially expressed
FDR: False discovery rate
FPKM: Fragments Per Kilobase of transcript per Million mapped reads
GLGB: Starch branching enzyme
GLGL: ADP-glucose pyrophosphorylase
GO: Gene ontology
GPT: Glucose-6-phosphate translocator
IDD5: Protein indeterminate-domain 5
PGMP: Phosphoglucomutase
RNA-seq: RNA sequencing
SSG: Granule-bound starch synthase
SSY: Starch synthase
TMM: Trimmed Mean of M-values
UGPA: UDP-glucose pyrophosphorylase
WOX4: WUSCHEL-related homeobox 4
